# Conversion among photo-oxidative products of polypropylene in solid, liquid and gaseous states

**DOI:** 10.1186/s13065-020-00698-y

**Published:** 2020-07-18

**Authors:** Xuan Liu, Rui Yang

**Affiliations:** Department of Chemical Engineering, Tsinghua University, Beijing, 100084 People’s Republic of China

**Keywords:** Polypropylene, Photo-oxidative aging, Conversion, Aging degree

## Abstract

During aging of polymers, oxidized species on macromolecular chains in solid state, volatile degradation products in liquid state and gaseous degradation products in gaseous state are often investigated separately. The conversion among these products is not especially concerned and biased results may be obtained based on the products in a single state. In this paper, photo-oxidative products of commercial polypropylene (PP) and unstabilized PP in solid, liquid and gaseous states were investigated by using Fourier transform infrared spectroscopy (FTIR), pyrolysis–gas chromatography/mass spectrometry (Py-GC/MS) and gas chromatography (GC). By comparing the formation profiles, conversion among the photo-oxidative products in three states was traced. During photo-oxidative aging, the main chains of PP were first oxidized to form carbonyl species in solid state, or fractured to form volatile alkenes as liquid. With the proceeding of aging, the oxidized main chains fractured to form small molecules, resulting in the conversion of oxidized species from solid state to liquid and gaseous states. When the aging degree was extremely high, the accumulation of liquid oxidized products was limited due to migration and condensation. Therefore, both the carbonyl index (CI) and the concentrations of volatile oxidized products were increased first and then decreased, while the concentrations of gaseous products kept increasing all along.

## Introduction

During the aging process of polymers, oxidation and degradation of macromolecular chains happened, leading to the deterioration of mechanical properties [1–3]. The aging products of polymers exist in solid, liquid and gaseous states. Solid products are resulted from the oxidation of main chains [4, 5]. Liquid products are small molecular degradation products, which can be absorbed in the aged polymers or volatilize [6]. Gaseous products are smaller molecules, which are in gaseous state at room temperature and easily migrate out [7]. Oxidized main chains continuously fractured to generate liquid and gaseous molecules. The dynamic process results in continuous change of products in three states. Therefore, studying the products in a single state is not enough to reflect the whole aging process.

Most researches focused on the changes of solid polymers before and after aging. For instance, changes of functional groups, chromophores, molecular weight, crystallinity and morphology were often used to evaluate the aging degree of polymers [8–12]. Some researchers turned their eyes to small molecular degradation products [13]. Carlsson, Wiles and Philippart contributed to the identification of volatile and gaseous products of polypropylene (PP) in various aging conditions [14–16]. Combination of selective isotopic labeling with solid-phase microextraction or cryotrapping gas chromatography/mass spectroscopy made it possible to confirm the original position of carbons on the main chains entering the volatile products [17]. Egerton et al. [18–21] analyzed the generation of CO_2_ in real time during photo-oxidation of a series of polymers, such as acrylic paint, low density polyethylene (LDPE), poly(vinyl chloride) and polyethylene terephthalate.

When the stabilities of polymers were compared based on macromolecular products or small-molecular degradation products separately, there might be conflicting results. For example, rare volatile oxidized products were generated during photo-oxidation of high density polyethylene composites, but the carbonyl index (CI) of the solid sample was rather high [22]. Another example was found in LDPE nanocomposites. LDPE/ZnO nanocomposites had lower CI but much higher concentration of CO_2_ than LDPE/TiO_2_ nanocomposites after photo-oxidative aging [23]. These facts remind us that the products in a single state can only provide information related to aging properties from a single aspect. However, there are few works on the whole aging procedure, including changes of solid, liquid and gaseous states together. In order to understand aging mechanism comprehensively, studying the conversion among aging products in three states is of great importance.

In this paper, photo-oxidative products of two PPs with different stabilities in solid, liquid and gaseous states were studied. Different induction periods indicated different generation times of these products. Conversion among the products in three states was traced. Overview of the aging products in three states was an effective way to obtain comprehensive information of the whole aging process and avoid possible biased evaluation of the aging degree and relative stability of polymers.

## Experimental

### Materials

Two kinds of PPs were used: commercial PP pellets (F401, purchased from Sinopec, with stabilizers’ package containing UV stabilizers and/or antioxidants, marked as CPP) and unstabilized PP powders (supplied by PetroChina Panjin Petrochemical Company, without any additives, marked as UPP). Both pellets and powders were hot-pressed at 190 °C into films (thickness of 0.3 mm).

### Photo-oxidative aging

**Fig. 1:**
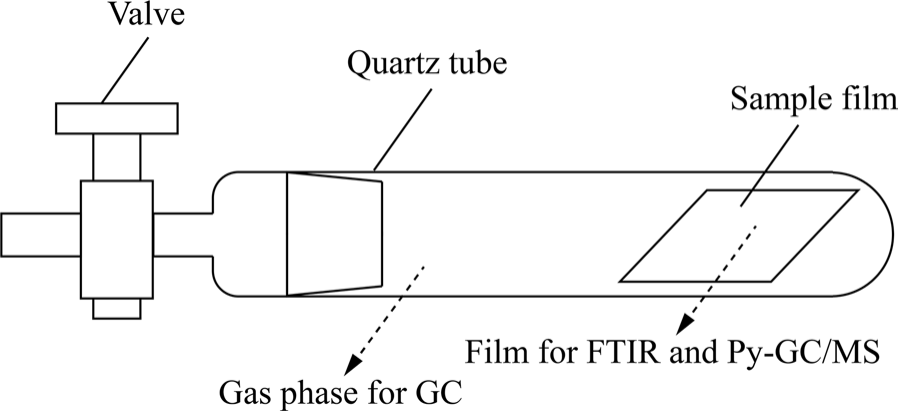
Schematic diagram of reaction device

Photo-oxidative aging was conducted in a Q-Sun Xe-3 chamber (Q-Lab Corporation). A film (40 mm × 15 mm) was put into a sealable quartz tube before aging (Fig. 1). The intensity of UV irradiation was 0.35 W/m^2^/nm@340 nm. The temperature was 60 °C and the time intervals were 48, 96, 144, 288, 384 and 480 h. Before aging, the valve was closed to seal the tube. After aging, the valve was opened and the gas in the tube was extracted for the gas chromatography (GC) measurement. Then the film was taken out for the Fourier transform infrared spectroscopy (FTIR) and pyrolysis-gas chromatography/mass spectrometry (Py-GC/MS) measurements.

### FTIR measurement

FTIR measurement was conducted to detect the oxidized macromolecules in solid state. Transmission spectra were obtained by using a Thermo-Nicolet iS10 FTIR spectrometer. For attenuated total reflection (ATR) mode, an accessory with a diamond crystal was used. CI was calculated by dividing the peak area at 1714 cm^−1^ to the peak area at 2722 cm^−1^ [24]. Three measurements were carried out for each film and the results were averaged.

### Py-GC/MS measurement

Py-GC/MS measurement was conducted in a gas chromatography/mass spectrometer (GC/MS-QP2010 SE, Shimadzu) installed with an EGA/PY-3030D multi-shot pyrolyzer (Frontier Laboratories) to detect the volatile degradation products absorbed by the film. 10.0 mg film or ethanol solution of the condensed liquid droplets on the tube wall was heated at 300 °C for 30 s and the evaporated components went through an Ultra Alloy-5 column. Limited by the stationary phase of the column, components with carbon number less than six might not be separated completely. The peak area of a component in the flash evaporation-gas chromatogram was calculated to represent the relative concentration of the component. Two measurements were carried out for each film and the results were averaged.

### GC measurement

GC measurement was conducted in a 7890B GC system (Agilent Technologies) to detect the gaseous degradation products around the film in the tube. 200 μL gas were extracted from a tube by an injector and injected into the GC system, through a G3591-81023 column. According to the separation capacity of the column, only small gaseous molecules like H_2_, N_2_, O_2_, CO, CO_2_, and alkanes and alkenes with carbon number less than three could be detected. A flame ionization detector and a thermal conductivity detector were used. The peak area of a component in the gas chromatogram was calculated to represent the relative concentration of the component.

## Results and discussion

### Macromolecular products

CI mainly reflected the accumulation of carbonyl products on the macromolecular chains in solid state. CI calculated from ATR spectra showed the local oxidation degree on the surface of the film, while CI calculated from transmission spectra showed the average oxidation degree in the bulk polymer.

**Fig. 2:**
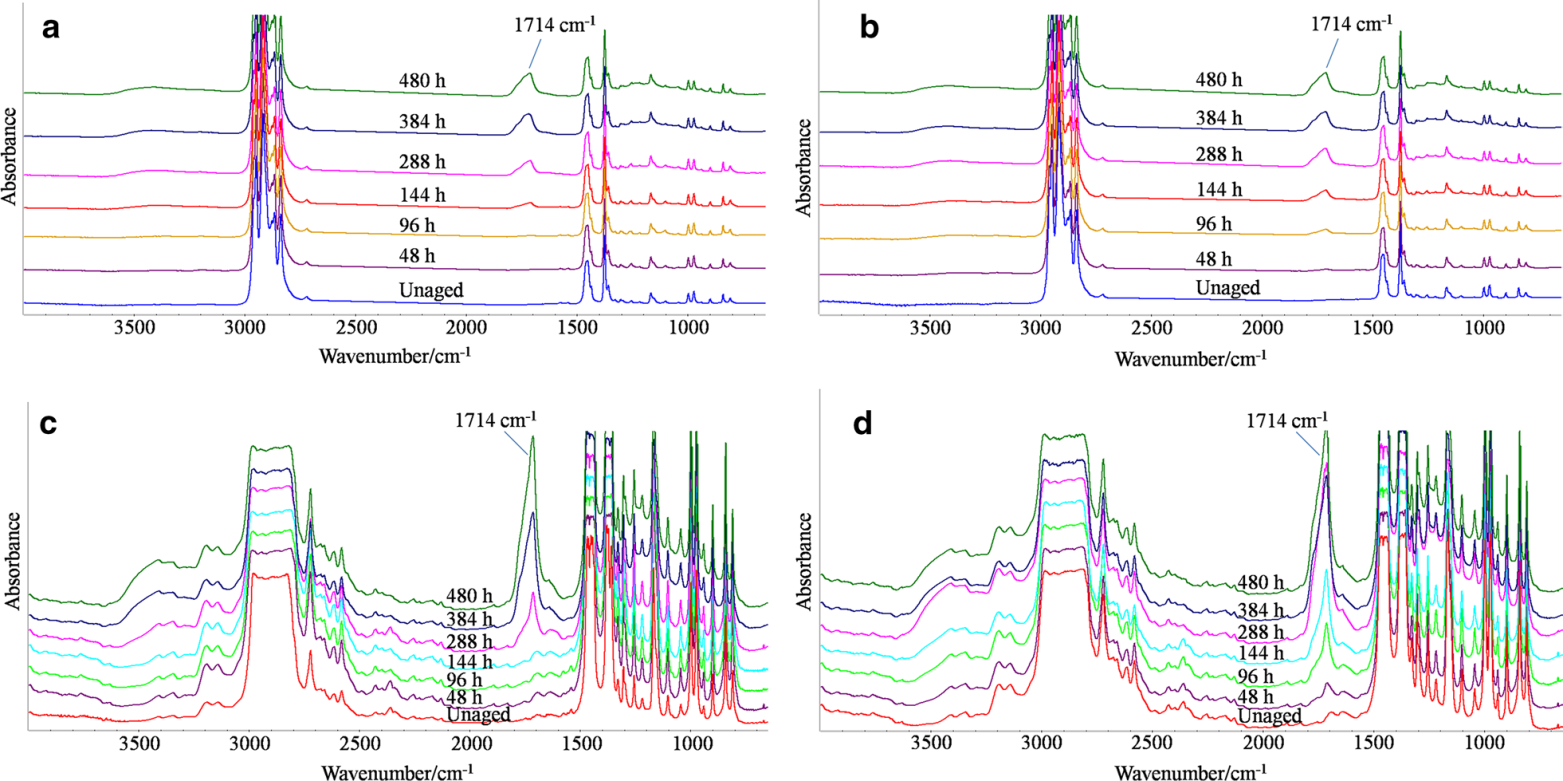
Infrared spectra of CPP and UPP during aging. **a** ATR spectra of CPP; **b** ATR spectra of UPP; **c** transmission spectra of CPP; **d** transmission spectra of UPP)

**Fig. 3:**
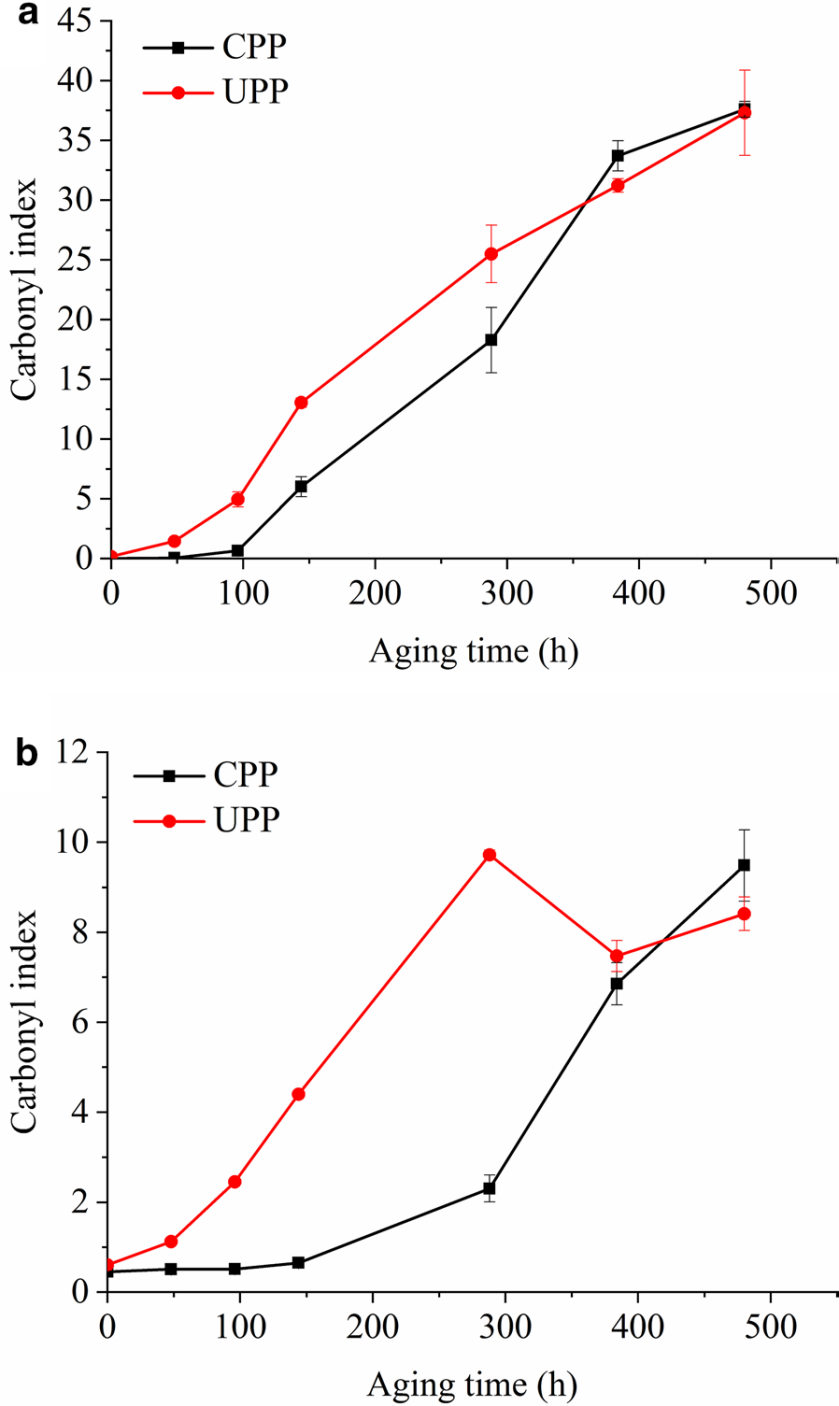
Carbonyl index of CPP and UPP with aging time. **a** calculated from ATR spectra; **b** calculated from transmission spectra

ATR spectra, transmission spectra and corresponding CI of two PPs are shown in Figs. 2 and 3. On the surface (Figs. 2a, b and 3a), CPP had an induction period of at least 96 h, while UPP was oxidized from the very beginning of aging. After the induction period, CPP was oxidized at a rate (0.10 h^−1^) similar to UPP (0.11 h^−1^) before 288 h. After that, the increase of CI in UPP slowed down. Two PPs seemed to reach the similar oxidation degree after 480 h. In the bulk (Figs. 2c, d and 3b), UPP was also oxidized from the very beginning of aging, while CPP exhibited an induction period of at least 96 h. The oxidation rate of CPP after 288 h (0.04 h^−1^) was the same to that of UPP before 288 h (0.04 h^−1^). After 288 h, the CI of UPP decreased.

The decrease of the CI in Fig. 3b seemed unreasonable, since the oxidation of PP was an accumulation process of the oxidized species. Therefore, some highly oxidized species might be lost. Considering that UPP was easier to be oxidized than CPP, it reached a high oxidation degree first. At the late stage of aging (after 288 h), severely oxidized fragments might drop from the surface and cause the decrease of CI [25–27]. The change of CI of UPP in Fig. 3a supported the speculation. The surface oxidation degree of UPP after aging for 288 h also showed a lower increasing rate than that of CPP. In this case, although the aging degree of UPP was evidently higher than that of CPP, the CI of UPP was close to or even lower than that of CPP after 288 h. Thus, the biased evaluation of the aging degree was obtained.

In addition, different sensitivities of ATR spectra and transmission spectra were observed. Despite the same difference between the oxidation degrees of two PPs after the same aging time, the differences between CI of two PPs were not the same from ATR spectra and transmission spectra. For instance, after aging for 288 h, the CI from ATR spectra of UPP was about 1.4 times of that of CPP, while the CI from transmission spectra of UPP was about 4.2 times of that of CPP. Obviously, transmission spectra were more sensitive to the different oxidation degrees.

### Volatile degradation products

**Fig. 4:**
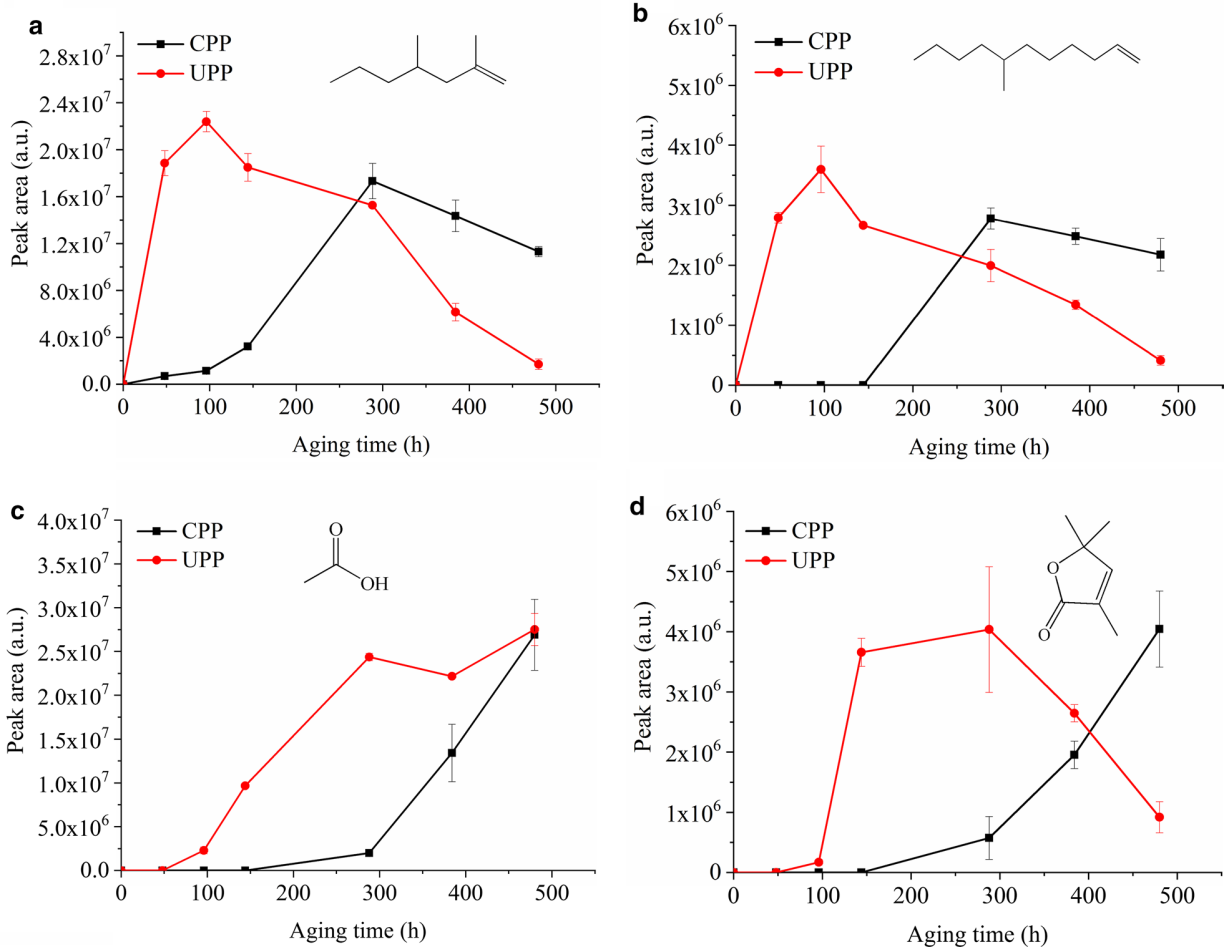
Peak area of four typical volatile degradation products in CPP and UPP with aging time. **a** and **b** two alkenes; **c** acetic acid; **d** lactone

Volatile degradation products were absorbed in PP film as liquid. They could be desorbed through flash evaporation and detected by Py-GC/MS. Four typical volatile products, i.e. two alkenes, acetic acid and a lactone, were identified in the flash evaporation-gas chromatograms of two PPs. The peak areas of these products with aging time are shown in Fig. 4. In Fig. 4a, b, the alkenes were generated from the very beginning of aging in UPP, while later than 96 h in CPP. After a period of time, i.e. 96 h in UPP and 288 h in CPP, the peak areas of two alkenes turned to decreasing, corresponding to the increasing of two oxidized products in Fig. 4c, d at the same time. This indicated that: (1) The formation of alkenes was prior to the formation of oxidized products, in other words, the chain scission of PP happened before the formation of volatile oxidized products; (2) The oxidized products might be from the further oxidation of alkenes.

**Fig. 5:**
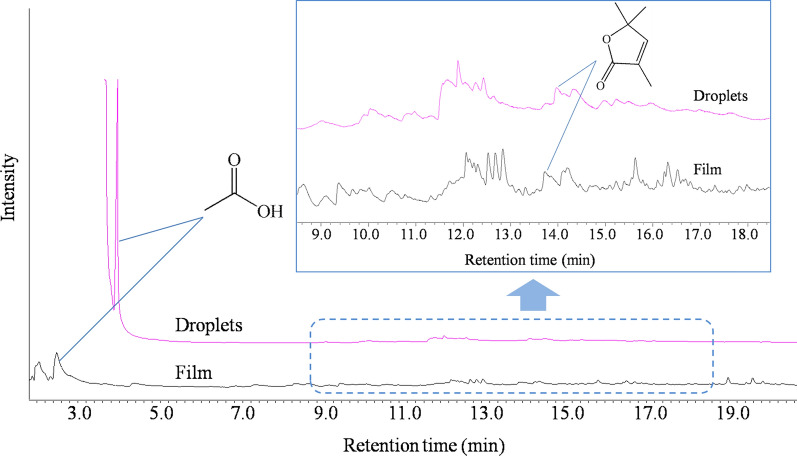
Flash evaporation-gas chromatograms of UPP and corresponding droplets in the tube after aging for 384 h

The peak areas of volatile oxidized products were expected to keep increasing with aging time due to the continuous oxidation of polymer chains. In CPP, they kept increasing indeed. In UPP, however, the peak areas stopped increasing and even began to decrease from 288 h. This was caused by the evaporation of these products and then condensation on the tube wall. There were visible droplets on the tube wall in which UPP was aged for 384 and 480 h. The droplets were washed by ethanol and the obtained solution was analyzed by Py-GC/MS. The flash evaporation-gas chromatogram of the droplets was compared with the result of the corresponding film (Fig. 5). Acetic acid and the lactone were identified in both the droplets and the corresponding film. Once the volatile oxidized products were evaporated and condensed on the tube wall, they could not come back to the film, so the peak areas in UPP were decreased obviously (Fig. 4c, d).

For CPP, no visible droplets were observed even after aging for 480 h, due to the relative low concentration of volatile oxidized products. There was a maximal concentration of volatile oxidized products that could be retained in the film. If the concentration did not exceed the maximum, the volatile oxidized products would accumulate in the solid polymer. When the concentration exceeded the maximum, the excessive parts began to be desorbed and some condensed on the tube wall. In this case, the biased evaluation of the aging degree of two PPs according to volatile degradation products would also be obtained as according to macromolecular products. Similar phenomenon can be expected if the polymer is photo-oxidized in unsealed atmosphere like in practical use.

### Gaseous degradation products

**Fig. 6:**
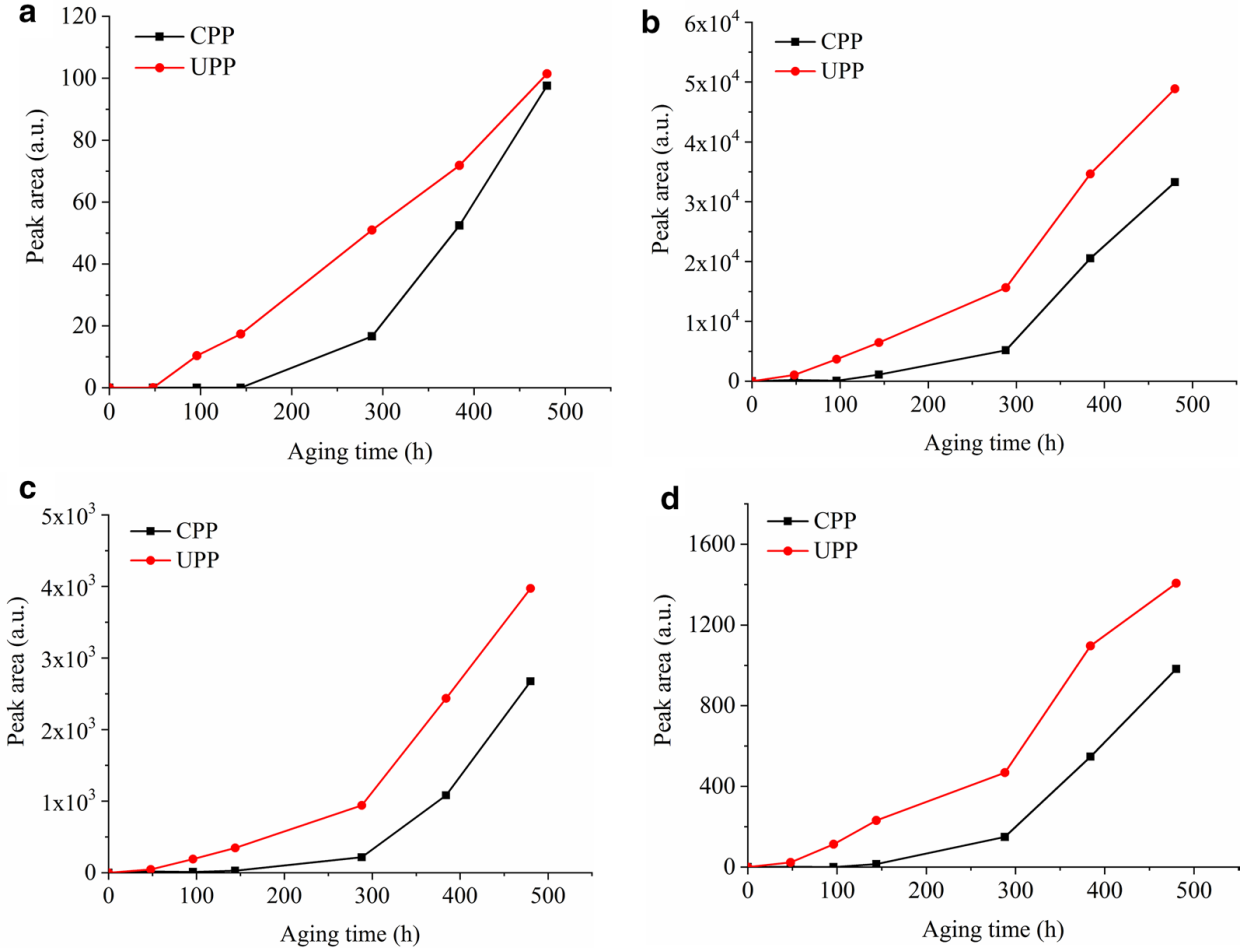
Peak area of four typical gaseous degradation products in CPP and UPP with aging time. **a** H_2_; **b** CO; **c** CH_4_; **d** C_2_H_4_

Gaseous degradation products included H_2_, CO, CO_2_, alkanes and alkenes. They were in gaseous state and could be identified directly by using GC measurement. The peak areas of four typical gaseous products with aging time are shown in Fig. 6. These products were generated from 48 h in UPP and from 144 h in CPP respectively. After that, the peak areas kept increasing in both PPs and the peak areas in UPP were higher than in CPP all along, showing the higher aging degree of UPP without the protection of stabilizers.

### Overview of photo-oxidative products in three states

Induction periods of the generation of photo-oxidative products in three states were extracted from Figs. 3, 4 and 6, listed in Table 1. In CPP, the induction periods of carbonyl products and volatile alkenes were the same, shorter than acetic acid, the lactone and gaseous degradation products, indicating the earlier generation of oxidation species in macromolecular chains and volatile alkenes. Thereafter volatile alkenes might be further oxidized and the oxidized main chains fractured to generate volatile oxidized products and gaseous degradation products. As shown in Figs. 4c, d and 6, the peak areas of acetic acid, the lactone and gaseous degradation products were increased rapidly from 288 h. At the same time, the peak areas of volatile alkenes were decreased rapidly (Fig. 4a, b). This phenomenon suggested the further oxidation of volatile alkenes and the corresponding accumulation of volatile oxidized products and gaseous degradation products.

**Table 1:** Induction period of generation of photo-oxidative products in CPP and UPP

Products	Induction period in CPP/h	Induction period in UPP/h
CI on the surface (solid)	96	0
CI in the bulk (solid)	96	0
Alkenes (liquid)	96	0
Acetic acid (liquid)	144	48
Lactone (liquid)	144	48
H_2_, CO, CH_4_, C_2_H_4_ (gas)	144	48

Compared with CPP, UPP exhibited much shorter induction periods. Without the protection of stabilizers, oxidation species in macromolecular chains and volatile alkenes were generated from the very beginning of aging, followed by volatile oxidized products and gaseous degradation products within 48 h. As shown in Figs. 3b, 4c, d, CI and the peak areas of volatile oxidized products were decreased at the late stage of aging, due to the loss of severely oxidized species in solid state and the migration and condensation of the liquid products. The peak areas of gaseous degradation products kept increasing for as long as 480 h.

**Fig. 7:**
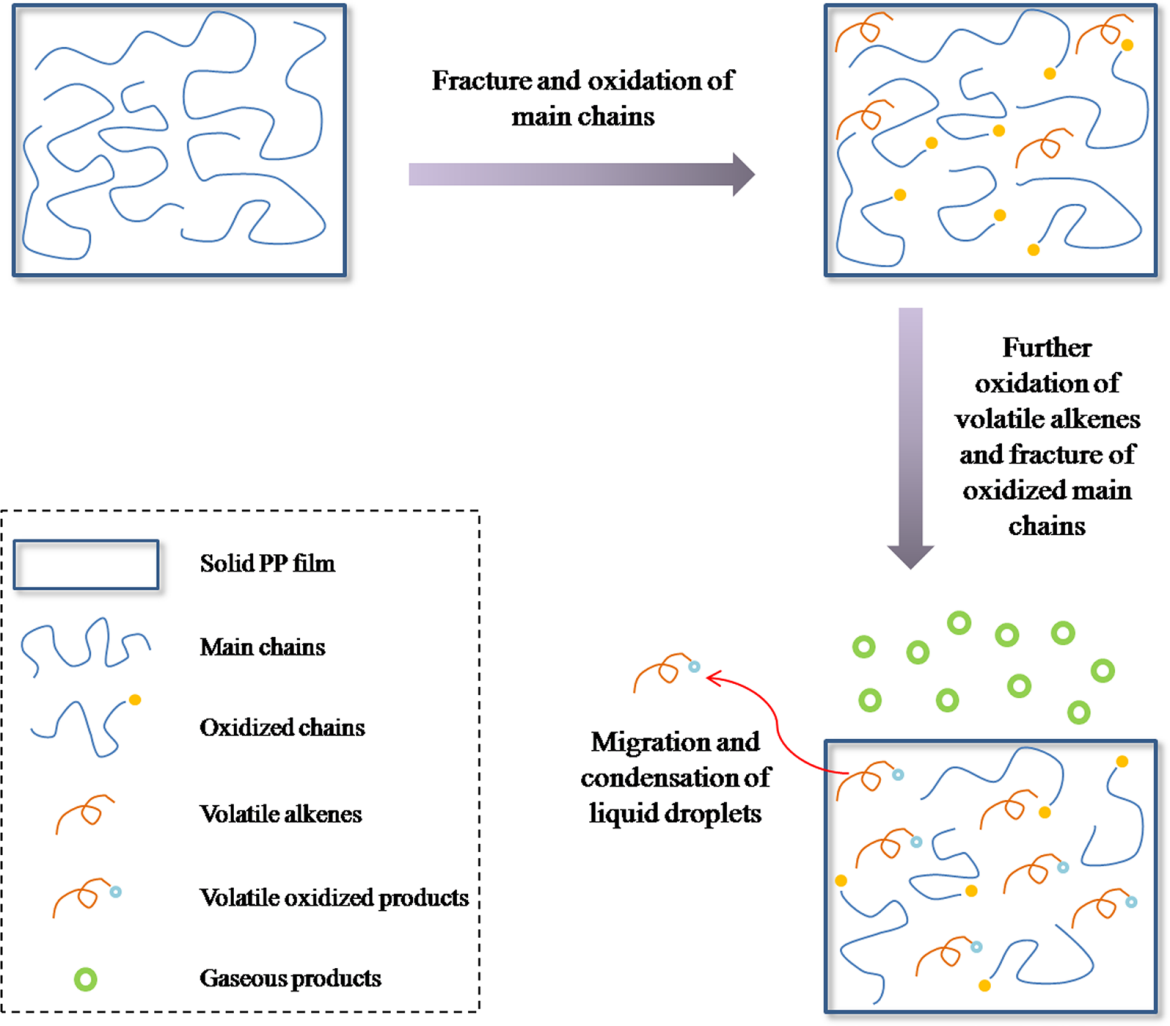
Schematic diagram of conversion among photo-oxidative products of PP in three states

Conversion among photo-oxidative products in three states is illustrated in Fig. 7. During the photo-oxidation of PP, the main chains were first oxidized and the oxidized species remained in the film as solid products. In the meanwhile, the main chains fractured to generate volatile alkenes, absorbed by the film as liquid. Then the oxidized main chains fractured along with the further oxidation of volatile alkenes, to generate volatile oxidized products absorbed by the film as liquid and gaseous degradation products in the atmosphere around the film. The conversion took place throughout the aging process. When the conversion was severe as in UPP, the CI and the concentrations of volatile products were increased first and then decreased, due to the loss of severely oxidized species in solid state and the migration and condensation of liquid droplets in the late stage, despite the continuous aging of the polymer. In CPP, the similar conversion was observed, although the aging process was retarded owing to the presence of stabilizers. Therefore, the products in a single state, especially only in solid state, could not reflect the comprehensive aging process and might lead to biased results. It was more reliable to consider the photo-oxidative products in three states as a whole when evaluating the aging degree and relative stability of polymers.

## Conclusions

The photo-oxidative products of CPP and UPP in solid, liquid and gaseous states were detected by FTIR, Py-GC/MS and GC respectively, and their formation profiles were compared. During photo-oxidative aging, the carbonyl products in macromolecular chains and volatile alkenes were generated first. Then the volatile oxidized products and gaseous degradation products were generated from the further oxidation of volatile alkenes and the fracture of oxidized main chains. At the late stage of aging, CI and the concentrations of volatile oxidized products were decreased, due to the loss of severely oxidized species in solid state and the migration and condensation of liquid droplets, while the concentrations of gaseous degradation products maintained increasing. Overview of the aging products in three states provides comprehensive information and overall understanding of the aging mechanism. It offers an effective way to evaluate the aging degree and relative stability of polymers accurately.

## Data Availability

The datasets used and/or analyzed during the current study are available from the corresponding author on reasonable request.

## Abbreviations

PP: Polypropylene
CPP: Commercial PP
UPP: Unstabilized PP
FTIR: Fourier transform infrared spectroscopy
ATR: Attenuated total reflection
Py-GC/MS: Pyrolysis–gas chromatography/mass spectrometry
GC: Gas chromatography
CI: Carbonyl index
